# Oropharyngeal Cartilaginous Choristoma Presenting as a Unilateral Mass

**DOI:** 10.1007/s12105-026-01917-z

**Published:** 2026-04-28

**Authors:** Imran Ajmal, Harishanker Jeyarajan, Melad N. Dababneh

**Affiliations:** 1 Department of Pathology, Heersink School of Medicine, University of Alabama at Birmingham, NP3545, 619 19th St S, Birmingham, AL 35249 USA; 2 Department of Otolaryngology, Head and Neck Surgery, Heersink School of Medicine, University of Alabama at Birmingham, Birmingham, AL USA

**Keywords:** Cartilaginous choristoma, Chondroid metaplasia, Chondroma, Chronic tonsillitis, Oropharynx

## Abstract

Oropharyngeal cartilaginous choristomas are uncommon incidental lesions identified in tonsillectomy specimens. We present an unusual example of a mass-forming cartilaginous choristoma of the palatine tonsil and share its macroscopic and histomorphologic features.

Cartilaginous choristomas (CC) are rare benign lesions of the head and neck, comprised entirely or predominantly of ectopic benign chondroid tissue [1]. While tumoral presentations are seen in the oral cavity, particularly the dorsal tongue, they are only an incidental subcentimetric and likely underreported finding in the oropharynx [1–5].

This is a case of a young adult male with chronic and recurrent tonsillitis, tonsilloliths, and a slowly growing tonsillar lesion over a 2-years period. Physical examination was notable for unilateral oropharyngeal fullness and a firm mass occupying one palatine tonsil and anterior tonsillar pillar, with a focal central defect and purulent discharge. Imaging revealed an enlarged tonsil with punctate calcifications, raising concern for neoplasm.

A biopsy revealed benign tonsillar mucosa and fragments of unremarkable hyaline cartilage. Subsequent bilateral tonsillectomy was performed. Macroscopic inspection and dissection of the affected tonsil showed a submucosal, well-defined, and multilobulated firm tan-white tumor, measuring 3.2 cm in greatest dimension (Figs. 1A, 2A). Histologically, the lesion was well-demarcated with irregular borders and composed entirely of benign mature hyaline cartilage without cytologic atypia, multinucleation, mitotic activity, or tumor-type necrosis. No ductal/epithelial or abluminal/myoepithelial component was identified, ruling out a stroma-rich pleomorphic adenoma. The chondroid tissue was embedded within fibrous stroma, focally adjacent to minor mucinous salivary gland lobules (Figs. 1B, 2A). The cartilage was frequently enveloped by a thin perichondrium-like layer (Fig. 2B) and in some areas, associated with collagen fibers (Fig. 2C), granulation tissue and acute inflammation (Fig. 2D), or microcalcifications and non-specific giant cell reaction (Fig. 2E). Interestingly, the contralateral tonsil had follicular lymphoid hyperplasia and a small focus of CC with a minute mature adipose tissue component (Fig. 3A, B). The patient had an uneventful postoperative recovery.

**Fig. 1:**
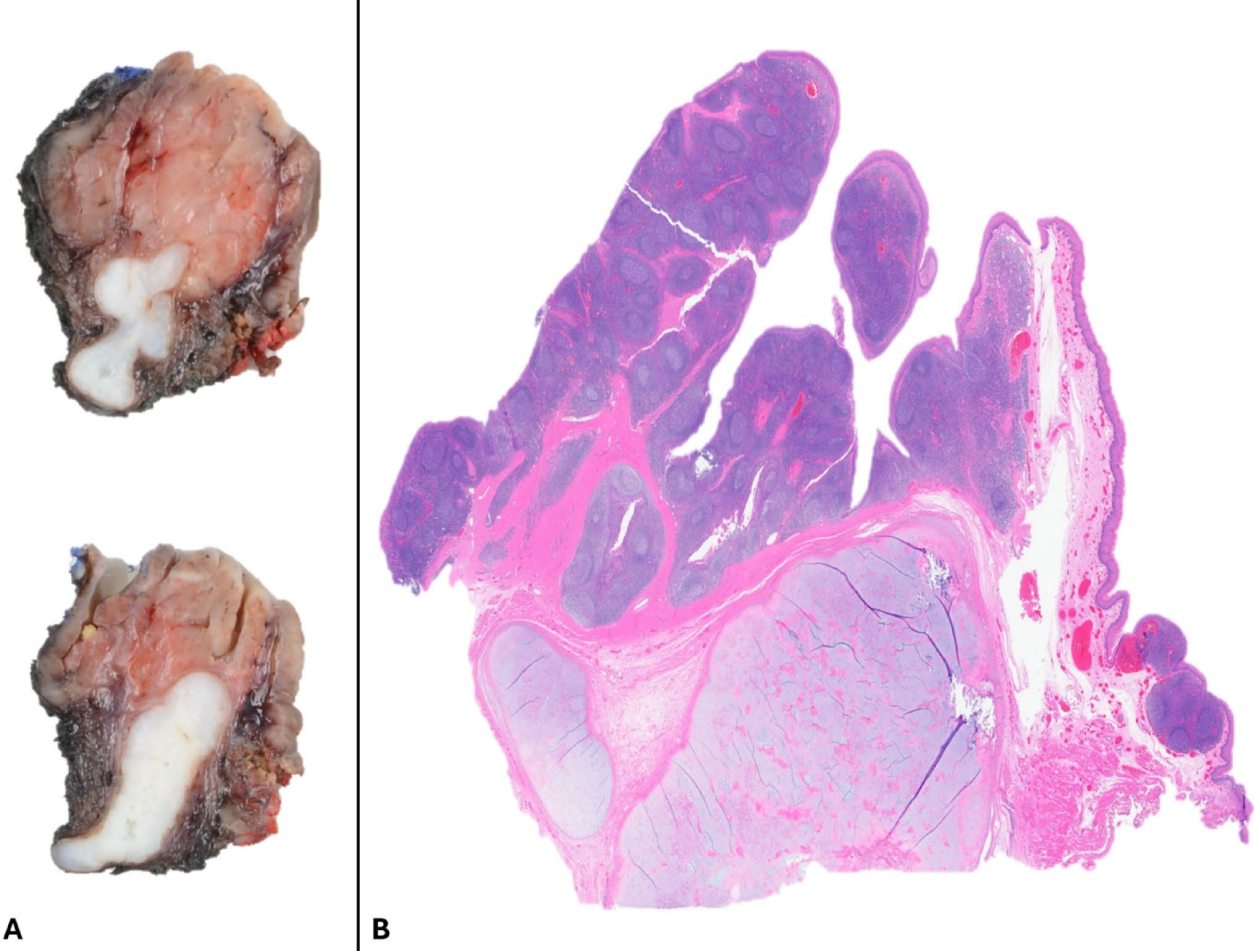
Macroscopic/gross appearance of the cartilaginous choristoma (**A**), and its corresponding microscopic appearance (**B**) presenting as a well-demarcated tan-white firm tissue, underlying the tonsillar crypts, and comprised of benign mature cartilage

**Fig. 2:**
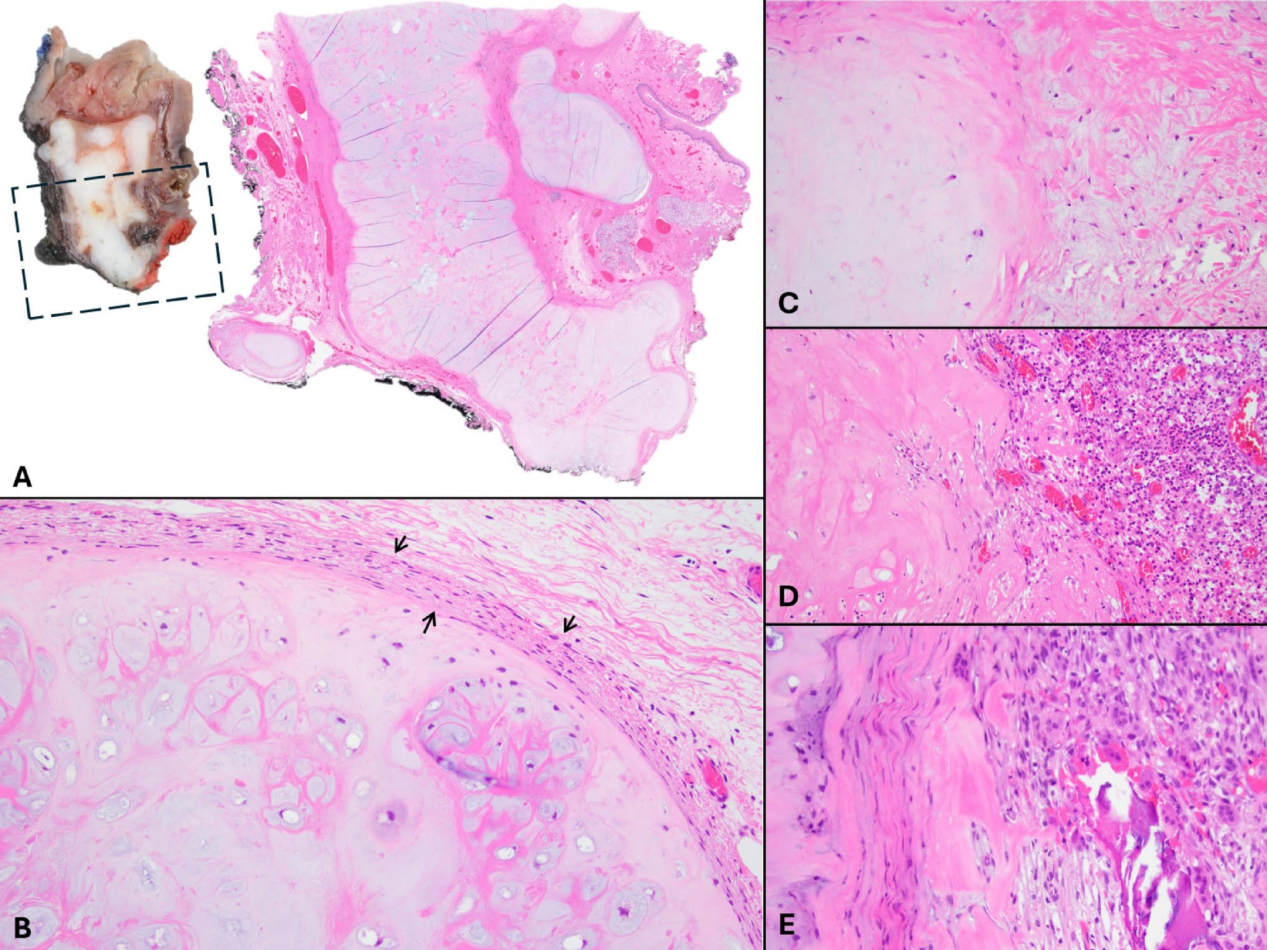
Cartilaginous choristoma appears as well-demarcated but multilobulated submucosal chondroid deposits with surrounding fibrosis (**A**, gross and corresponding microscopic image). The hyaline cartilage displays a variety of interactions with the surrounding tissue, including areas of perichondrium-like peripheral cellular condensation (**B**, black arrows), wisps of pink fibrous tissue/collagenous material (**C**, right half), granulation tissue and mixed acute and chronic inflammation (**D**, right half), and focal mineralization/calcification with giant cell reaction (**E**, right half)

**Fig. 3:**
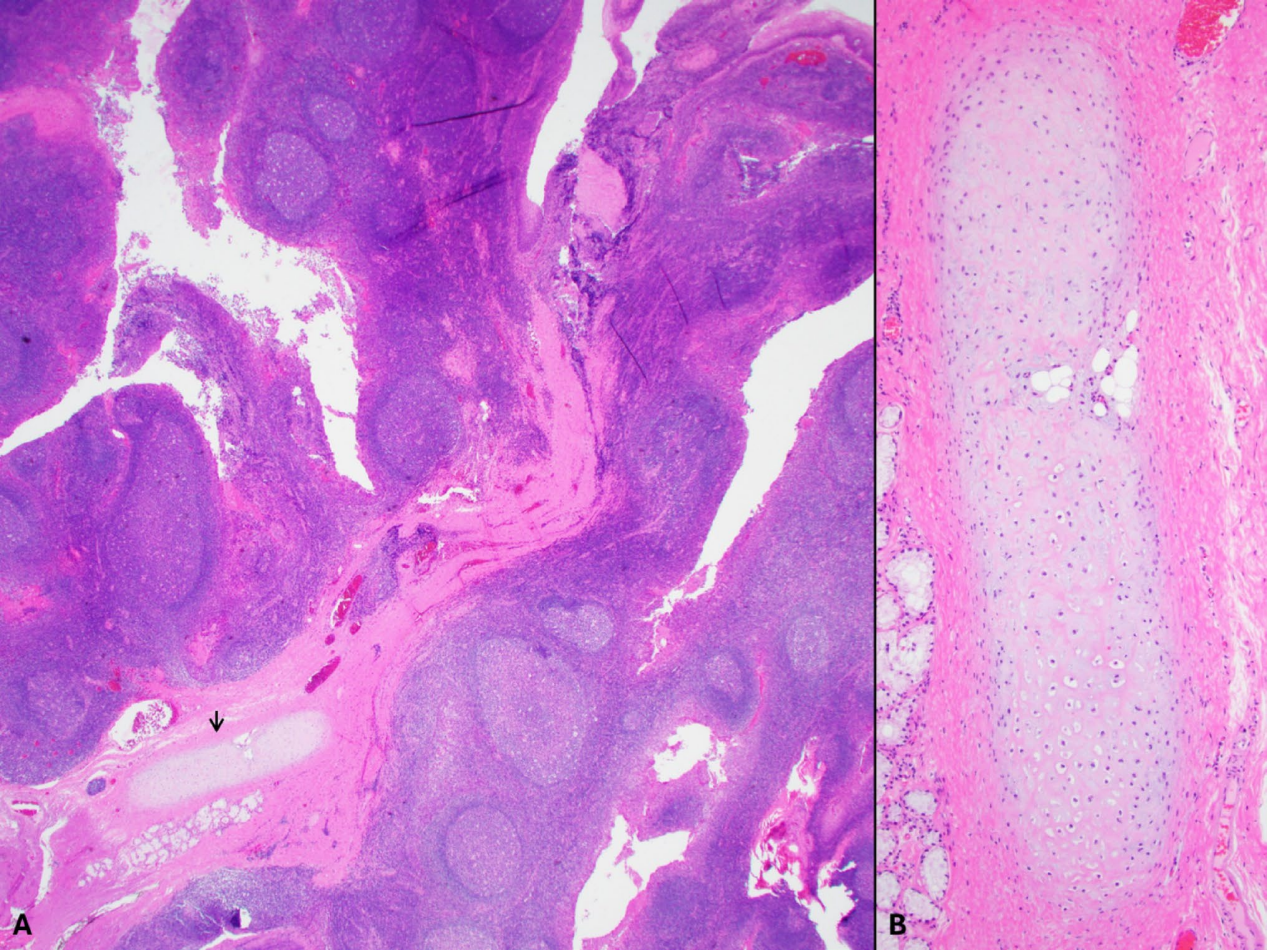
The contralateral hyperplastic tonsil has an incidental small focus of mature hyaline cartilage with minute lobules of adipose tissue, present within fibrous septation, consistent with cartilaginous choristoma (**A**, **B**). This represents the more common presentation of these lesions

CCs are typically an incidental microscopic finding in tonsillectomy specimens. To our knowledge, this is the first example of a mass-like CC of the palatine tonsil, and it is the largest reported to date at this anatomic site. The presence of ectopic cartilage in the other tonsil suggests that both lesions represent different stages of the same pathological process.

## Data Availability

No datasets were generated or analysed during the current study.
